# Self-monitoring of oral anticoagulation: does it work outside trial conditions?

**DOI:** 10.1136/jcp.2008.059634

**Published:** 2009-01-20

**Authors:** C Gardiner, I Longair, M A Pescott, H Erwin, J Hills, S J Machin, H Cohen

**Affiliations:** 1 Department of Haematology, University College London Hospitals, London, UK; 2 Department of Haematology, University College London, London, UK

## Abstract

**Background:**

Patient self-monitoring (PSM) of oral anticoagulation therapy (OAT) can improve anticoagulant control, but poor uptake and high dropout rates have prompted suggestions that PSM is suitable for only a minority of patients in the UK.

**Aims:**

To determine whether PSM could be a viable alternative to regular hospital anticoagulant clinic attendance, if offered from the start of treatment.

**Methods:**

318 consecutive patients referred, for the first time, to an anticoagulation clinic were assessed for eligibility using established criteria. Patients electing for PSM attended training and, following successful assessment, performed a capillary blood INR every two weeks or more frequently if directed to do so by the anticoagulation clinic. Primary outcome measures were uptake of PSM and the percentage time in target therapeutic INR range (TIR) compared to patients electing for routine clinic care.

**Results:**

Of 318 patients referred for OAT, 188 were eligible for PSM. 84 (26%) elected to self-monitor, of whom 72 (23%) remained self-monitoring or had completed their course of treatment at the end of the audit. Self-monitoring patients had significantly better anticoagulant control than those receiving routine hospital anticoagulation clinic care (TIR 71% vs 60%, p = 0.003) and significantly less time outside critical limits, ie, INR <1.5 or >5.0 (0.45% vs 2.04%, p = 0.008).

**Conclusions:**

Patients offered PSM from the start of treatment show increased uptake compared to previous UK studies and a level of oral anticoagulation control comparable to that reported in previous clinical trials.

An estimated 950 000 people in the UK receive oral anticoagulant therapy (OAT) and the numbers are increasing at approximately 10% per year. The main driver for this expansion is the increasing use of OAT for stroke prevention in patients with atrial fibrillation, where less than 25% of patients are thought to be receiving OAT.^1^

Several studies in the UK have shown the quality of care achieved by patient self-monitoring of oral anticoagulation to be at least as good as that attained by hospital or primary care anticoagulation clinics.^2^^–^7 However, these studies used only selected patients who were already established on warfarin and managed in anticoagulation clinics for several months prior to enrolment. Furthermore, the uptake of self-monitoring was highly variable (10–41%) and the dropout rate in most studies has been high (9–42%),^8^ prompting suggestions that self-monitoring of OAT is suitable for only a minority of patients. A recent review of studies performed in the UK suggested that only 14% of all eligible patients would conduct long-term self-monitoring of UK OAT.^1^ In the majority of studies, the total population from which patients were selected was not stated, making it difficult to assess uptake in absolute terms. In our previous studies,^2^ ^4^ where, as was the case in other UK studies, self-monitoring was offered to patients established on oral anticoagulation, we found that many patients were discouraged by the clinical trial setting, rather than self-monitoring per se. Others were reassured by the familiarity of the hospital anticoagulation clinic, and were reluctant to change to self-monitoring for this reason. We also identified non-compliance as a cause of poor anticoagulation control and increased dropout rates.

In the UK, patients should expect to be within their own target therapeutic range for at least 60% of the time^9^ and, therefore, this is also the standard that any alternative model of OAT management should achieve. It has been suggested that an improvement of 10% in time in therapeutic range over routine care is required if a method is to be considered superior.^3^ Previous studies conducted in the UK have demonstrated percentage time in therapeutic range (%TIR) of 61–71% for patients who were self-testing only, and 70–76% for patients who were self-testing and self-dosing (ie, patient self-management).^1^

The aim of this audit was to determine whether self-monitoring of oral anticoagulation from the start of treatment and outside trial conditions, is acceptable to patients and a viable alternative to regular attendance at the anticoagulant clinic.

## PATIENTS AND METHODS

The study was a prospective closed cohort audit of 318 consecutive patients referred for oral anticoagulation between July 2005 and March 2007. These patients, who had not previously received oral anticoagulation, were assessed for suitability for self-monitoring, using established criteria,^10^ ie, patients with known drug or alcohol abuse, atypical INR target ranges, anticipated short therapy duration physical or intellectual impairment, which would preclude self-monitoring, and patients with language barriers unless a named carer or interpreter was available. No other assumptions were made about the patients’ ability to self-monitor. The Joint UCL/UCLH Committees on the Ethics of Human Research deemed this an audit of clinical practice and, as such, ethical approval was not required.

### Protocol

Patients who expressed a desire to self-monitor attended a nurse-led training course, comprising two sessions, which covered effects of diet, medication and alcohol on warfarin, and the use of a point-of-care (POC) monitor (CoaguChek S, Roche Diagnostics, Basel, Switzerland). Following a successful assessment, patients were then asked to perform a capillary blood INR once every two weeks. They contacted the anticoagulation clinical nurse specialists (CNS) with their INR result by phone and were advised by the CNS of dose changes and their next test date. Dosing was performed by the CNS using computer assisted dosing (4S DAWN Clinical Software, Milnthorpe, UK). CNS were permitted to override the computerised decision support software. The manufacturer’s internal quality control was performed as per UK guidelines^10^ and external quality control was achieved by six-monthly parallel testing,^11^ in which the patient’s POC device was compared to a reference POC device. Evidence of non-compliance was investigated and retraining given if required. Those patients who did not want to self-monitor, or who had still not made a decision after 2 months, remained under the care of the routine hospital anticoagulation clinic. All patients were asked to report any problems with bruising, bleeding or thrombosis. A major bleed was defined as that requiring hospitalisation and/or blood transfusion.

After a suitable period of familiarisation (at least 6 weeks), self-monitoring patients were asked if they would like to self-manage their OAT. Those expressing an interest were given additional training in dose-adjustment using an algorithm supplied by the clinic. After a successful assessment, these patients commenced self-management (ie, self-testing and self-dosing of OAT). All patients undertaking self-monitoring sign a contract with the Trust which includes agreement to undertake self-monitoring as specified by the anticoagulation clinic, when to contact the clinic, when to undertake quality control (QC) checks (with QC preparations supplied by the Trust), and for review and external quality assessment.

### Analysis

Percentage TIR was calculated using the method of linear interpolation, described by Rosendaal *et al*.^12^ Data from the first 6 weeks of treatment were not included in the %TIR analysis, as OAT control is known to be poor during this period.^13^ Patients with unusually narrow therapeutic target ranges were excluded from all analyses. All patients with less than five INR results (after the initial six-week period) were also excluded from the %TIR analysis. The proportions of INR tests within, below and above the therapeutic range were also recorded. As the risks of thrombosis and bleeding are known to increase exponentially at INRs <1.5 and >5.0 respectively,^14^ the incidence of INR values outside these critical limits was also calculated. The data showed non-normal distribution, so two-tailed non-parametric methods were used; the Wilcoxon signed rank test was used to test for differences between median values for paired data, and the Mann–Whitney U test was used for independent groups. A p value of <0.05 was considered statistically significant.

## RESULTS

**Figure 1:**
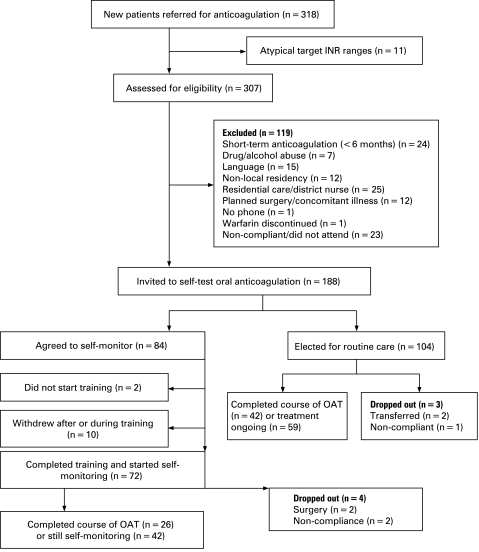
Study protocol.

In total, 188/318 consecutive patients referred for oral anticoagulation for the first time, were eligible for self-monitoring. Eleven patients were excluded from all further analysis because they had non-standard target INR ranges. Another 119 patients were judged to be ineligible for self-monitoring according to criteria set out in the British Committee for Standards in Haematology^10^ guidelines. Of the 188 eligible patients, 84 (44%) agreed to self-monitor while 104 declined or failed to make a decision within two months of their first appointment (fig 1).

Of the remaining 68/84 (81%) patients, 26 completed their course of treatment and 42 were still self-monitoring at the end of the 20-month audit period. Four patients had fewer than five self-monitoring results because the duration of treatment was shorter than anticipated; these data were therefore excluded from the time in therapeutic range analysis. Of the 104/188 patients electing for routine anticoagulation clinic care, only 88 had five or more data points after the initial six-week period and only these 88 were included in the statistical analysis. Self-monitoring patients were significantly younger than those electing for routine care (58 vs 68 years of age, p<0.001) and the increased proportion of patients with atrial fibrillation receiving routine care reflected this. Men and women were distributed equally between the treatment groups (table 1).

**Table 1:** Patient demographics

	Number or median	
	Self-test	Routine care
Male (n)	43	54
Female (n)	41	51
Median age (years) (95% CI)	58* (53.6 to 63.0)	68 (63.0 to72.0)
Indications for anticoagulation		
Atrial fibrillation	32	59
Replacement heart valve	8	3
Venous thromboemboli	36	40
Cardiovascular prophylaxis	7	1
Other	1	2
Target INR range		
1.5–2.5	1	2
2.0–3.0	72	100
2.5–3.5	4	1
3.0–4.0	7	2

*p<0.001.

### Therapeutic INR control

The median %TIR was significantly higher in the self-monitoring patients than in those receiving routine anticoagulation clinic care (71% vs 60%, p = 0.003). In those patients who had been self-monitoring (n = 28) or receiving routine care (n = 27) for at least one year, the %TIR showed a further improvement compared with the %TIR in patients receiving routine anticoagulation clinic care (%TIR 74% (95% CI 69.9 to 80.6) vs 64% (95% CI 60.8 to 69.7), p = 0.01) (table 2). In the patients who elected to self-manage, the median %TIR was 74% (95% CI 65.6 to 82.2).

**Table 2:** Percentage time in target therapeutic range

	Self-test (n = 67)	Routine care (n = 88)	p Value
Median % time in therapeutic range	71 (64.1 to 75.3)	60 (55.0 to 63.2)	0.003
% of tests below therapeutic range	22	30	<0.0001
% of tests in therapeutic range	58	49	
% of tests above therapeutic range	20	21	

The median time between tests was 11 days (range 4–22) in the self-testing patients and 18 days (range 5–45) in the patients receiving routine care. When the percentage times outside critical limits (INR <1.5 or >5.0) were studied, patients who monitored their own INR showed a significant reduction in the amount of time that they were outside these limits when compared to those patients receiving routine anticoagulation clinic care (median 0.45% vs 2.04%, p = 0.008) (table 3).

**Table 3:** Percentage time outside critical control limits (INR <1.5 or >5.0)

	Self-monitor (n = 67)	Routine care (n = 88)	p Value
Median % time outside critical control range	0.45 (0.0 to 1.8)	2.04 (0.68 to 4.34)	0.008
% of tests below critical limit	3.4	9.0	<0.0001
% of tests within critical limits	93.3	88.8	
% of tests above critical limit	3.3	2.2	

### Adverse events

We had 59.3 patient-years of follow-up data for the self-monitoring patients, during which there was one major bleed, five minor bleeds and two cases of thrombosis. One patient died prior to training due to an unrelated medical condition. The incidence of major bleeds was 1.7 per 100 patient-years, minor bleeds 8.4 per 100 patient-years and thrombosis 3.4 per 100 patient-years. We had 73.9 patient years of follow-up for the patients receiving routine care, during which there were four major bleeds (requiring blood transfusion or hospitalisation), eleven minor bleeds and one case of thrombosis. The incidence of major bleeds was 5.4 per 100 patient-years, minor bleeds 16.2 per 100 patient-years and thrombosis 1.4 per 100 patient-years.

## DISCUSSION

We have audited the acceptability and efficacy of self-monitoring of OAT, when offered at the start of initial anticoagulant treatment. Other than to exclude patients for whom self-monitoring was inappropriate, recruitment was non-selective; 26% of all patients (44% of eligible patients) agreed to self-monitor. Most published studies used the number of eligible patients as the denominator, making it difficult to accurately assess total uptake, but we believe that our uptake of 26% represents an improvement on previous UK studies.^1^ As with previous studies, a significant number of patients dropped out during or shortly after training (14%), but most of the patients who started self-monitoring completed their course of treatment or continued to self-monitor after the end of the audit period (94%). A recent systemic review^1^ reported that only 14% of eligible patients in the UK would conduct long-term monitoring of OAT, whereas 36% of eligible patients referred to our anticoagulation clinic were able and willing to self-monitor when offered self-monitoring at the start of anticoagulation. As with previous studies, self-monitoring patients were younger than the clinic population as a whole,^1^ but we found no gender-related difference in uptake.

The %TIR for self-monitoring patients in this audit is similar to that observed in previous studies in the UK^2^^–^7 and a recent study performed in the Netherlands,^15^ and superior to that in a large Spanish study.^16^ Most other studies of self-monitoring of OAT were performed in countries where specialised hospital anticoagulant clinics are not normal clinical practice. Consequently, it is not appropriate to discuss these studies here.

While the uptake and retention of patients in our audit were superior to those in previous studies performed in the UK and the Netherlands (where anticoagulation is usually undertaken in specialist hospital-based clinics), recruitment was higher in the Spanish study^16^ and is typically much higher (60–70%) in Germany, where self-monitoring is widely practiced.^17^ The reasons for this are not entirely clear, but reimbursement and patient motivation have been cited as possible reasons.^3^

This audit had several limitations. As this was an audit of a new service provision, no randomisation was performed. The patients who elected for routine anticoagulation clinic care were older than the self-monitoring patients and they may have been predisposed to poorer OAT control. The difference in testing intervals between the two groups makes comparison of %TIR problematic and the shorter interval between testing may have been contributory to the higher %TIR in the self-monitoring patients.

Self-monitoring of OAT has been shown to reduce the incidence of thrombotic and haemorrhagic complications^18^ and it may enhance the quality of life in some patients including those who travel frequently, who are in employment or education, and who find it difficult to travel to clinics.^1^ Although this study was not powered to assess the rate of adverse events, our data showed a trend towards reduced bleeding and thrombotic events in self-monitoring patients. While it is unlikely that self-monitoring of OAT will become cost effective compared to specialist anticoagulant clinics in the UK,^1^ the National Institute for health and Clinical Excellence recommends that patients with atrial fibrillation who require long-term anticoagulation should be considered for self-monitoring if preferred by the patient and eligibility criteria are met.^19^ Test-strips for self-monitoring are now available on the Drug Tariff, but several barriers to self-monitoring remain. Quality control (QC) preparations are not available on prescription and some patients are reluctant to perform QC, highlighting the importance of addressing the supply of QC preparations and a contract with the Trust specifying when QC testing should be undertaken. Many GPs refuse to prescribe test-strips and some Primary Care Trusts refuse to fund self-monitoring. The General Medical Services Contract National Enhanced Service is open to interpretation on issues surrounding funding of self-monitoring, which may limit progress on its implementation. Some patients see the price of the monitors as a major obstacle to self-monitoring.

We conclude that self-monitoring is acceptable to many patients if offered at the start of treatment and it is also efficacious. Our data suggest that a quarter of unselected patients referred for oral anticoagulation choose self-monitoring; they achieve a quality of oral anticoagulation comparable to that reported in previous UK clinical trials.

**Take-home messages**

- Self-monitoring offered at the start of oral anticoagulation is associated with improved uptake and fewer dropouts.
- Outside trial conditions, self-monitoring patients achieve a quality of anticoagulant control, which may be superior to that attained in routine specialist anticoagulation clinics.
